# Cognitive and Affective Determinants of Academic Success Among Female Freshmen in a Society Emerging from Patriarchy

**DOI:** 10.1192/j.eurpsy.2026.11959

**Published:** 2026-07-31

**Authors:** S. Aamir, M. Pilotti, M. Bo Julaia

**Affiliations:** Core Humanities & Social Sciences, Prince Mohammad Bin Fahd University, Al Khobar, Saudi Arabia

## Abstract

**Introduction:**

Student populations from the Global South, particularly female students from societies transitioning away from patriarchal norms, remain underrepresented in mental health research. In such contexts, first-year university women face unique cognitive and emotional demands as they navigate new academic challenges while countering persistent gender stereotypes. Understanding how these factors interact is vital for informing culturally sensitive mental health support.

**Objectives:**

This study aimed to examine how cognitive difficulties (e.g., self-reported memory lapses), affective factors (e.g., anxiety), and personal strengths (e.g., self-efficacy and prior achievement) relate to first-year academic success among Saudi female freshmen. It further explored whether these relationships differ between students enrolled in male-dominated STEM fields and non-STEM programs.

**Methods:**

A purposive sample of 1,141 Saudi female freshmen (STEM: n=482; non-STEM: n=659) enrolled at an English-medium university participated (97% response rate). Emotional difficulties were measured using the GAD-7; cognitive challenges via the Prospective and Retrospective Memory Questionnaire (PRMQ); self-efficacy through the General Academic Self-Efficacy Scale (GASE); and prior achievement with high school GPA. First-year GPA indexed academic performance. ANOVA and linear regression analyses tested group differences and predictors of GPA. Ethical approval was obtained from the institution’s Deanship of Research (PMU-DoR-2023-2024-17).

**Results:**

Non-STEM students reported significantly higher anxiety levels (M=11.75 vs. 10.53; *p*<.001), more prospective memory lapses (*p*=.034), and lower high-school GPAs (*p*=.001) than STEM peers, but no difference emerged in first-year GPA. Regression analyses showed that for non-STEM students, higher memory lapses and lower self-efficacy predicted lower first-year GPA. For STEM students, greater anxiety related to school issues and more memory lapses predicted lower GPA. Notably, anxiety in non-STEM students did not significantly impact their academic attainment, suggesting possible compensatory mechanisms via self-efficacy.

**Image 1:**
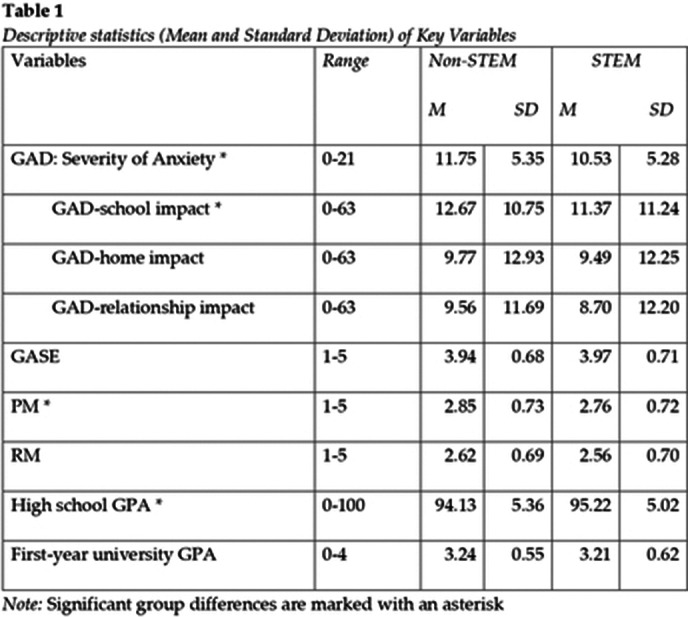
Image 1: Long description.

**Image 2:**
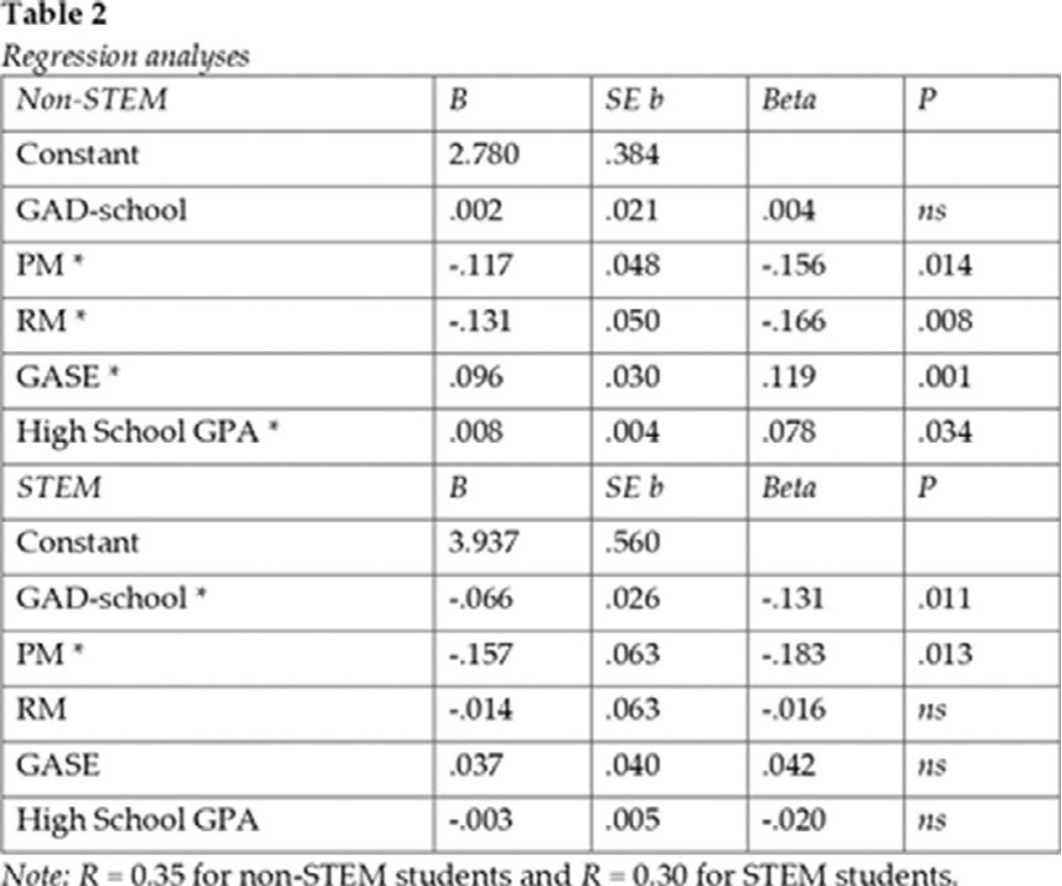
Image 2: Long description.

**Conclusions:**

Findings underscore how sociocultural transitions shape students’ mental health and academic adjustment. While non-STEM students reported greater anxiety, this did not translate into poorer achievement, possibly due to stronger self-efficacy buffering effects. Conversely, anxiety negatively impacted STEM students’ performance, highlighting the persistent influence of gender stereotypes in male-dominated fields. Elevated anxiety and low self-efficacy may signal risks for psychiatric outcomes, including depression, warranting targeted mental health support. Interventions should address both individual coping and systemic barriers, such as gender bias in STEM, to foster resilience and success among underrepresented student groups.

**Disclosure of Interest:**

S. Aamir Employee of: None., M. Pilotti Employee of: None., M. Bo Julaia Employee of: None.

## Long descriptions

### Image 1: Long description

The table contains six columns: Variables, Range, Non-S T E M M, Non-S T E M S D, S T E M M, and S T E M S D.

* G A D Severity of Anxiety asterisk: Range 0 to 21. Non-S T E M: M 11.75, S D 5.35. S T E M: M 10.53, S D 5.28.

* G A D school impact asterisk: Range 0 to 63. Non-S T E M: M 12.67, S D 10.75. S T E M: M 11.37, S D 11.24.

* G A D home impact: Range 0 to 63. Non-S T E M: M 9.77, S D 12.93. S T E M: M 9.49, S D 12.25.

* G A D relationship impact: Range 0 to 63. Non-S T E M: M 9.56, S D 11.69. S T E M: M 8.70, S D 12.20.

* G A S E: Range 1 to 5. Non-S T E M: M 3.94, S D 0.68. S T E M: M 3.97, S D 0.71.

* P M asterisk: Range 1 to 5. Non-S T E M: M 2.85, S D 0.73. S T E M: M 2.76, S D 0.72.

* R M: Range 1 to 5. Non-S T E M: M 2.62, S D 0.69. S T E M: M 2.56, S D 0.70.

* High school G P A asterisk: Range 0 to 100. Non-S T E M: M 94.13, S D 5.36. S T E M: M 95.22, S D 5.02.

* First year university G P A: Range 0 to 4. Non-S T E M: M 3.24, S D 0.55. S T E M: M 3.21, S D 0.62.

A note at the bottom states that significant group differences are marked with an asterisk.

Navigate back to Image 1:.

### Image 2: Long description

The table is divided into two main sections.

Section 1: Non-S T E M. Columns are B, S E b, Beta, and P.

- Constant: B 2.780, S E b .384.

- G A D-school: B .002, S E b .021, Beta .004, P n s.

- P M asterisk: B -.117, S E b .048, Beta -.156, P .014.

- R M asterisk: B -.131, S E b .050, Beta -.166, P .008.

- G A S E asterisk: B .096, S E b .030, Beta .119, P .001.

- High School G P A asterisk: B .008, S E b .004, Beta .078, P .034.

Section 2: S T E M. Columns are B, S E b, Beta, and P.

- Constant: B 3.937, S E b .560.

- G A D-school asterisk: B -.066, S E b .026, Beta -.131, P .011.

- P M asterisk: B -.157, S E b .063, Beta -.183, P .013.

- R M: B -.014, S E b .063, Beta -.016, P n s.

- G A S E: B .037, S E b .040, Beta .042, P n s.

- High School G P A: B -.003, S E b .005, Beta -.020, P n s.

A note at the bottom states that R equals 0.35 for non-S T E M students and R equals 0.30 for S T E M students.

Navigate back to Image 2:.

